# Identification of the Regulatory Logic Controlling *Salmonella* Pathoadaptation by the SsrA-SsrB Two-Component System

**DOI:** 10.1371/journal.pgen.1000875

**Published:** 2010-03-12

**Authors:** Ana M. Tomljenovic-Berube, David T. Mulder, Matthew D. Whiteside, Fiona S. L. Brinkman, Brian K. Coombes

**Affiliations:** 1 Michael G. DeGroote Institute for Infectious Disease Research and the Department of Biochemistry and Biomedical Sciences, McMaster University, Hamilton, Canada; 2 Department of Molecular Biology and Biochemistry, Simon Fraser University, Burnaby, Canada; Universidad de Sevilla, Spain

## Abstract

Sequence data from the past decade has laid bare the significance of horizontal gene transfer in creating genetic diversity in the bacterial world. Regulatory evolution, in which non-coding DNA is mutated to create new regulatory nodes, also contributes to this diversity to allow niche adaptation and the evolution of pathogenesis. To survive in the host environment, *Salmonella enterica* uses a type III secretion system and effector proteins, which are activated by the SsrA-SsrB two-component system in response to the host environment. To better understand the phenomenon of regulatory evolution in *S. enterica*, we defined the SsrB regulon and asked how this transcription factor interacts with the *cis*-regulatory region of target genes. Using ChIP-on-chip, cDNA hybridization, and comparative genomics analyses, we describe the SsrB-dependent regulon of ancestral and horizontally acquired genes. Further, we used a genetic screen and computational analyses integrating experimental data from *S. enterica* and sequence data from an orthologous regulatory system in the insect endosymbiont, *Sodalis glossinidius*, to identify the conserved yet flexible palindrome sequence that defines DNA recognition by SsrB. Mutational analysis of a representative promoter validated this palindrome as the minimal architecture needed for regulatory input by SsrB. These data provide a high-resolution map of a regulatory network and the underlying logic enabling pathogen adaptation to a host.

## Author Summary

All organisms have a means to control gene expression ensuring correct spatiotemporal deployment of gene products. In bacteria, gene control presents a challenge because one species can reside in multiple niches, requiring them to coordinate gene expression with environmental sensing. Also, widespread acquisition of DNA by horizontal gene transfer demands a mechanism to integrate new genes into existing regulatory circuitry. The environmental awareness issue can be controlled using two-component regulatory systems that connect environmental cues to transcription factor activation, whereas the integration problem can be resolved using DNA regulatory evolution to create new regulatory connections between genes. The evolutionary significance of regulatory evolution for host adaptation is not fully known. We studied the convergence of environmental sensing and genetic networks by examining how the *Salmonella enterica* SsrA-SsrB two-component system, activated in response to host cues, has integrated ancestral and acquired genes into a common regulon. We identified a palindrome as the major element apportioning SsrB on the chromosome. SsrB binding sites have been selected to co-regulate a gene program involved in pathogenic adaptation of *Salmonella* to its host. In addition, our results indicate that promoter architecture emerging from SsrB-dependent regulatory evolution may support both mutualistic and parasitic bacteria-host relationships.

## Introduction

Precise gene regulation is crucial to the successful activation and execution of virulence programs for all pathogenic organisms. The acquisition of genes through horizontal gene transfer, a widespread means of bacterial evolution [1], requires a process to integrate new coding sequence into pre-existing regulatory circuitry. Silencing of horizontally-acquired genes by DNA binding proteins like H-NS is one way some incoming genes are initially controlled [2],[3], which can then be subject to regulatory evolution by mutating *cis*-regulatory operator regions to select for optional gene expression. The eventual promoter architecture selected to deploy virulence genes is often modular and should reflect a design that maximizes organismal fitness while limiting fitness trade-offs and antagonistic pleiotropy [4]. Both simulated [5] and functional experiments [6],[7] show that mutation of *cis-*regulatory sequences can be rapid, and that plasticity - the degree to which regulatory mutation can perturb the larger gene network - can be well tolerated in bacterial systems.

Promoter architectures that control quantitative traits such as bacterial virulence are, in fact, modular and evolvable [8]. For instance, *Salmonella enterica* has a multi-faceted pathogenic strategy fine-tuned by several transcriptional regulators. Intracellular survival and persistence of *Salmonella* requires a type III secretion system (T3SS) encoded in a horizontally-acquired genomic island called *Salmonella* Pathogenicity Island-2 (SPI-2) [9],[10]. T3SS are complex secretion machines that deliver bacterial effector proteins directly into host cells through an injectisome during infection [11],[12]. Several ancestral regulators control the genes in the SPI-2 genomic island including the two-component systems EnvZ-OmpR and PhoQ-PhoP, and the regulatory protein SlyA [13]. SsrA-SsrB is another two-component regulatory system co-inherited by genetic linkage with the SPI-2 locus that is essential for gene expression in SPI-2 [14]–[16]. SsrA is a sensor kinase activated in the host environment that phosphorylates the SsrB response regulator to create an active transcription factor needed for spatiotemporal control of virulence genes [13],[17]. In the *Salmonella* genus, the SPI-2 genomic island is found only in pathogenic serotypes of *Salmonella enterica* that infect warm-blooded animals and is absent from *Salmonella bongori*, which colonizes cold-blooded animals [18]. It is generally accepted that SPI-2 was acquired by *Salmonella enterica* after divergence from *S. bongori*, providing a useful pedigree to study regulatory evolution influenced by SsrB.

We recently demonstrated the evolutionary significance of *cis-*regulatory mutations for pathoadaptation of *Salmonella enterica* serovar Typhimurium (*S.* Typhimurium) to an animal host [19]. Our focus was on SsrB because of its broad conservation among the pathogenic Salmonellae and its essentiality for animal infection, suggesting it coordinates fundamental aspects of *Salmonella* pathogenesis beyond the SPI-2 genomic island. In this study we investigated how regulatory evolution assimilates horizontally acquired and ancestral genes into the SsrB regulon on a genome-wide scale using an integrated set of experimental methods. Combining our data with previous biochemical work, along with comparative genomic analyses with an orthologous T3SS-encoding genomic island in the tsetse fly endosymbiont, *Sodalis glossinidius*, we reveal the flexible DNA palindrome that distributes SsrB in the genome to influence transcriptional activation of the SPI-2 T3SS and almost all of its accessory effector proteins. Our data uncovers the principal SsrB circuitry that appears to have been conserved to support multiple bacterial lifestyles, including parasitic and mutualist symbioses.

## Results

### Transcriptional profiling of an *ssrB* mutant

**Figure 1:**
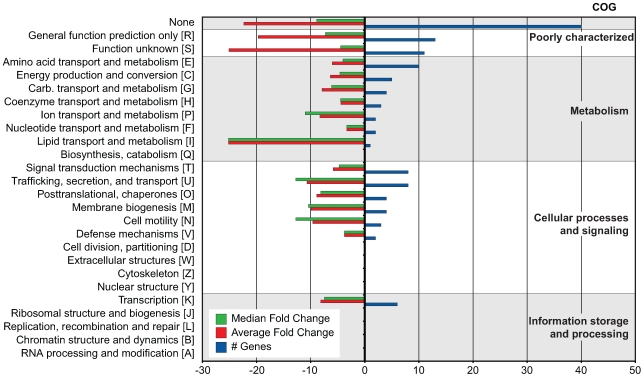
COG analysis of 133 genes co-regulated with SPI-2. COG categories are indicated to the right of the figure and COG sub-categories are ordered in terms of decreasing gene representation (blue) within each category. Median and average fold change values for each sub-category are indicated in green and red, respectively.

To begin to understand regulatory evolution and network expansion of the SsrB response regulator, we profiled the transcriptome of an *ssrB* mutant and compared it to *S.* Typhimurium wild-type cells grown in an acidic minimal medium that activates the SsrA-SsrB two-component regulatory system [20]. We identified 133 genes that were significantly down-regulated in the *ssrB* mutant [z <−1.96] [21] (Table 1). This included almost all genes in the SPI-2 genomic island as well as effector genes encoded throughout the genome (Dataset S1). Next, we performed a Clusters of Orthologous Groups (COG) analysis [22] on the 118 genes that had an ortholog in the annotated genome of *S.* Typhimurium strain LT2 [23]. Among these, 45 genes lacked a functional COG assignment and the 73 remaining genes were distributed among 86 COGs (Figure 1). The majority of functions in the latter groups are in transport, secretion, and trafficking of cellular components in addition to protein and membrane modification.

**Table 1:** Summary of microarray and ChIP-on-chip data.

Microarray	
Total significant SsrB-activated genes, *n*	133
Genes with *S. bongori* ortholog, *n*	47
Mean fold change	−15.8 (−6.0*)
Median fold change	−6.5 (−4.5*)
Genes within pathogenicity islands, *n*	45
Genes with *S. bongori* ortholog, *n*	2
Mean fold change	−27.4 (−6.5*)
Median fold change	−13.1 (−6.5*)
Genes within phage islands, *n*	4
Genes with *S. bongori* ortholog, *n*	0
Mean fold change	−20.4 (n/a*)
Median fold change	−9.0 (n/a*)
Genes within *S. bongori* ROD, *n*	7
Genes with *S. bongori* ortholog, *n*	0
Mean fold change	−14.6 (n/a*)
Median fold change	−7.1 (n/a*)
Genes not in genomic islands, *n*	77*^a^*
Genes with *S. bongori* ortholog, *n*	45
Mean fold change	−8.9 (−6.0*)
Median fold change	−4.4 (−4.4*)
**ChIP-on-chip**	
Probes, *n*	42,021
Probe size	60-bp
Average probe separation	57-bp
Genome probe average log_2_ signal	−0.02
Standard deviation	0.52
Peaks above 3 st. dev., *n*	256
Peaks within published islands, *n*	47
Peaks within *S. bongori* ROD, *n*	15
Within CDS, *n*	126
Within/Overlapping IGR, *n*	130

***n*** number.

*(value for genes with an ortholog in *S. bongori*).

***a*** includes 21 genes on the microarray that could not be mapped because they were not annotated by the array manufacturer (TIGR).

The SsrA-SsrB system was acquired by horizontal gene transfer into the *S. enterica* species after divergence from what is now extant *S. bongori*. As such, *S. bongori* has evolved in the absence of SsrA-SsrB and its regulatory architecture has not been influenced by it. Orthologous genes ancestral to both species but regulated by SsrB in *S. enterica* provide evidence for network expansion and regulatory evolution that we previously showed can be mapped to a single *cis*-input location by using functional and comparative genomics [19]. To expand on this, we used a reciprocal BLAST-based analysis and identified 47 orthologs in *S. bongori* among the 133 genes whose transcription was down-regulated in an *ssrB* mutant (Table 1). In Δ*ssrB* cells, the mean fold-change of the orthologous genes (−6.1-fold) was subtler than for the *S. enterica*-specific gene set (mean −21.2-fold), which included the T3SS and associated effector genes (Dataset S1). We also determined the distribution of down-regulated genes among genomic islands [24], including prophages, pathogenicity islands (SPI-islands) and additional regions of difference (ROD) between *S. enterica* and *S. bongori* (Dataset S2). For this we used a BLAST-based comparison of genome-wide synteny between *S.* Typhimurium and *S. bongori* and identified 50 ROD that included 17 previously reported SPI-islands and prophages. Of the 133 down-regulated genes identified, 56 were present within genomic islands (Table 1), with a mean change in gene expression of −25.3-fold in Δ*ssrB* cells.

### Genome-wide SsrB interactions

**Figure 2:**
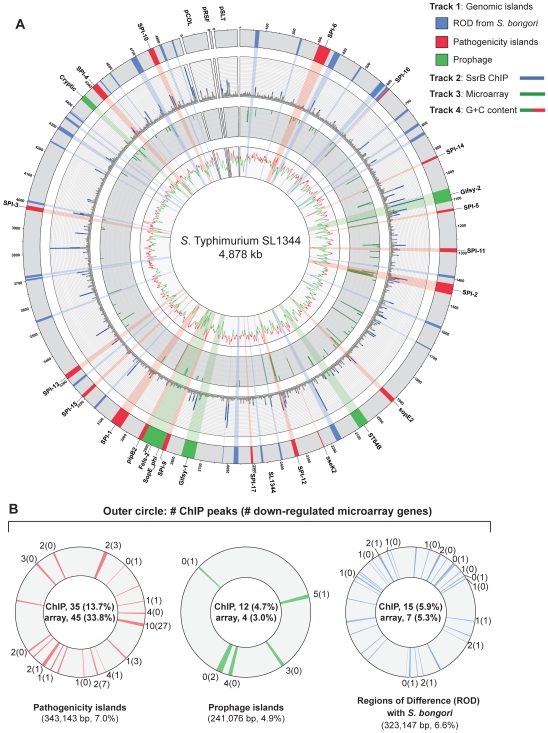
Genome-wide functional genomics analyses for SsrB. (A) ChIP-on-chip and microarray analysis. Genomic islands are indicated and labelled on track 1 (outermost circle), where prophages are in green, pathogenicity islands are in red and regions of difference (ROD) with *S. bongori* are in blue. ChIP-on-chip interaction scores (log_2_) for SsrB-activating conditions are plotted on track 2, where peaks with signals greater than one standard deviation from the mean probe signal are indicated in blue. Transcriptional profiling analysis of SsrB-regulated genes is plotted on track 3. Genes repressed more than 5-fold in an *ssrB* mutant are indicated in green. G+C content is plotted on track 4, where values greater than 53% (genome mean) are indicated in red and less than 53% are indicated in green. (B) Summary of gene expression data and SsrB binding data for pathogenicity islands, prophage islands, *S. bongori* ROD and genome sequence not in islands. The number of SsrB binding peaks identified by ChIP-on-chip for each of the individual islands is shown in the outermost track, along with the number of genes down-regulated in the transcriptional profiling analysis (in brackets). The total number of ChIP-on-chip interaction peaks and genes regulated by SsrB are shown in the innermost circles along with the percent of total (in brackets). The total genomic content of each island or conserved regions are shown below each circle, along with the percent of total genome. The remainder of microarray data (not in islands) includes 194 interaction peaks (75.8%) for ChIP-on-chip and 77 genes (58.0%) for transcriptional profiling arrays.

To examine SsrB allocation on the chromosome *in vivo*, we isolated functional SsrB-DNA interactions using chromatin immunoprecipitation and examined the bound DNA by chip analysis (ChIP-on-chip) using an *S.* Typhimurium SL1344 array containing 44,021 probes. With this method, we identified 256 significant interaction peaks distributed throughout the genome that were enriched under SsrB-activating conditions and with interaction scores three standard deviations greater than the mean probe score (Figure 2A and Table 1). Of these 256 peaks, 126 (49%) occurred within coding regions of genes (CDS) and 130 (51%) were in intergenic regions (IGR). Given the strong influence of SsrB on horizontally acquired genes (Table 1), we plotted the ChIP-on-chip data against all genomic islands in *S.* Typhimurium SL1344. From this analysis, 62 of 256 SsrB binding peaks (24.3%) occurred within genomic islands (Figure 2B).

SsrB ChIP peaks were observed upstream of previously identified SsrB regulated genes indicating that our ChIP-on-chip data captured functional interactions (Dataset S3). To generate a consensus set of SsrB regulated genes, we performed an analysis to identify operons in the *S.* Typhimurium SL1344 genome that encoded at least one gene down-regulated in Δ*ssrB* cells and that possessed an SsrB binding peak in the upstream regulatory region as defined by our ChIP-on-chip analysis. From this, the 133 down-regulated genes mapped to 86 operons, 49 of which had an SsrB interaction upstream or within the first gene of the operon (Table 2). This analysis captured all five reported operons in the SPI-2 genomic island in addition to ten operons outside of this island that encode SPI-2 translocated effectors.

**Table 2:** Predicted operons encoding SPI-2 associated genes.

Gene Range	Operon Size	Fold Change	First Gene	LT2 Ortholog	Annotation	Probe Location^a^	ChIP Score^b^
SL2601-SL2604	4	−1.45	SL2604	STM2640:*rpoE*		CDS	4.12
SL0991-SL0991	1	−57.81	SL0991	STM1051:*sseI*	Effector	IGR	3.37
SL2256-SL2256	1	−43.50	SL2256	STM2287:*sseL*	Effector	IGR	3.02
SL2217-SL2217	1	−7.07	SL2217	STM2241:*sspH2*	Effector	CDS	2.95
SL1561-SL1561	1	−25.20	SL1561	STM1631:*sseJ*	Effector	CDS	2.82
SL1628-SL1628	1	−51.60	SL1628	STM1698:*steC*	Effector	5′-IGR-overlap	2.48
SL1574-SL1576	3	−3.41	SL1576	STM1646:*ydbH*		CDS	2.40
SL1325-SL1326	2	−9.64	SL1326	STM1392:*ssrA*	SPI-2 gene	IGR	2.31
SL1327-SL1330	4	−18.09	SL1327	STM1393:*ssaB*	SPI-2 gene	IGR	2.31
SL1161-SL1161	1	−17.23	SL1161	STM1224:*sifA*	Effector	IGR	2.26
SL1340-SL1340	1	−10.51	SL1340	STM1406:*ssaG*	SPI-2 gene	CDS	1.93
SL0909-SL0909	1	−15.47	SL0909	STM0972:*sopD2*	Effector	5′-IGR-overlap	1.87
SL1027-SL1027	1	−33.97	SL1027	STM1088:*pipB*	Effector	IGR	1.85
SL1331-SL1336	6	−49.46	SL1331	STM1397:*sseA*	SPI-2 gene	IGR	1.81
SL1353-SL1356	4	−12.90	SL1353	STM1419:*ssaR*	SPI-2 gene	5′-IGR-overlap	1.78
SL1347-SL1352	6	−16.59	SL1347	STM1413:*ssaM*	SPI-2 gene	5′-IGR-overlap	1.76
SL1563-SL1566	4	−3.10	SL1563	STM1633		5′-IGR-overlap	1.76
SL2763-SL2763	1	−195.60	SL2763	STM2780:*pipB2*	Effector	IGR	1.75
SL0083-SL0083	1	−8.71	SL0083	STM0082:*srfN*		IGR	1.54
SL1908-SL1910	3	−2.68	SL1908	STM1979:*fliP*		3′-IGR-overlap	1.35
SL2001-SL2011	11	0.66	SL2011	STM2035:*cbiA*		IGR	1.30
SL4242-SL4245	4	−0.98	SL4242	STM4305.S		IGR	1.28
SL0700-SL0700	1	−7.06	SL0700	STM0719		CDS	1.18
SL3259-SL3261	3	−1.29	SL3261	STM3288:*yhbC*		IGR	1.13
SL1992-SL2000	9	−1.57	SL2000	STM2024:*cbiL*		CDS	0.96
SL2114-SL2114	1	−19.35	SL2114	STM2138:*srcA*	Effector	CDS	0.94
SL1026-SL1026	1	−2.64	SL1026	STM1087:*pipA*	Effector	IGR	0.90
SL1159-SL1160	2	−5.29	SL1160	STM1223:*potC*		CDS	0.86
SL1341-SL1346	6	−42.72	SL1341	STM1407:*ssaH*	SPI-2 gene	3′-IGR-overlap	0.84
SL1785-SL1785	1	−11.81	SL1785	STM1856		IGR	0.81
SL0037-SL0038	2	−4.00	SL0037	STM0036		5′-IGR-overlap	0.78
SL1170-SL1171	2	−2.84	SL1171	STM1233:*ycfC*		5′-IGR-overlap	0.74
SL1337-SL1339	3	−8.51	SL1337	STM1403:*sscB*	SPI-2 gene	3′-IGR-overlap	0.72
SL1878-SL1878	1	−7.65	SL1878	STM1949:*yecF*		IGR	0.72
SL1705-SL1705	1	−6.08	SL1705	STM1777:*hemA*		5′-IGR-overlap	0.69
SL2193-SL2196	4	−2.43	SL2193	STM2216:*yejA*		CDS	0.68
SL4478-SL4479	2	−1.83	SL4478	STM4547:*yjjQ*		IGR	0.67
SL1763-SL1763	1	−21.12	SL1763	STM1834:*yebN*		IGR	0.62
SL0758-SL0760	3	−6.10	SL0758	STM0781:*modA*		5′-IGR-overlap	0.60
SL1200-SL1200	1	−10.18	SL1200	STM1265		CDS	0.58
SL1238-SL1242	5	−1.54	SL1238	STM1303:*argD*		CDS	0.56
SL1958-SL1961	4	−26.21	SL1961	-		IGR	0.56
SL2210-SL2211	2	−19.29	SL2211	STM2235		CDS	0.56
SL0701-SL0703	3	7.94	SL0701	STM0719		5′-IGR-overlap	0.55
SL0547-SL0549	3	−5.97	SL0549	STM0559:*rfbI*		IGR	0.55
SL0393-SL0393	1	−3.20	SL0393	STM0398:*phoR*		CDS	0.53
SL1689-SL1692	4	0.78	SL1692	STM1764:*narG*		IGR	0.53
SL0160-SL0160	1	−3.75	SL0160	STM0159		IGR	0.52
SL1373-SL1375	3	−1.29	SL1375	STM1443:*ydhI*		IGR	0.52

^***a***^ CDS (within coding sequence); IGR (in intergenic region); 5′-IGR overlap (probe overlaps IGR and 5′ region of CDS).

^***b***^ log_2._

### SsrB binds to six promoters within the SPI-2 genomic island

**Figure 3:**
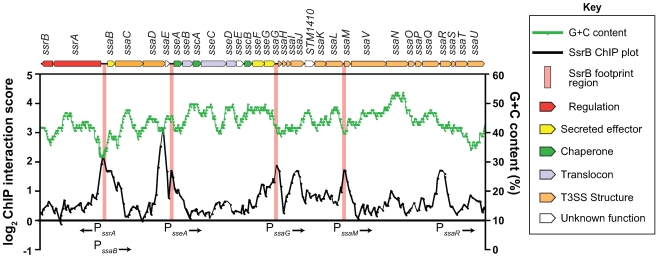
The SsrB binding profile at the SPI-2 locus. Plotted are the SsrB ChIP interaction scores (black trace) for all probes within the SPI-2 locus. ChIP-on-chip experiments were performed with *S*. Typhimurium grown in SPI-2 inducing (LPM) and non-inducing (LB) conditions. ChIP score was calculated as LB (control/experimental)/LPM(control/experimental) where LB scores were used to normalize the data. Regions of SPI-2 footprinted by SsrB are highlighted with vertical pink bars. G+C content across the region is plotted as a green trace. Genes in the SPI-2 genomic island (top) are plotted to scale and are coloured according to function (see key).

In order to rigorously evaluate our genome-wide functional genomics data, we compared it against traditional biochemical experiments describing SsrB-DNA interactions at the SPI-2 locus. Previous data reported SsrB footprints upstream of 6 genes in SPI-2: *ssrA, ssrB, ssaB, sseA, ssaG,* and *ssaM* [25],[26]. Our ChIP-on-chip data showed discrete SsrB binding at all of these promoters except for the promoter reported to be between *ssrA* and *ssrB* [25] (Figure 3). We attempted to verify functional activity at this site, but could not using transcriptional fusions (data not shown). Our data also identified three additional SsrB binding peaks upstream of *sseA*, within the CDS of *ssaJ*, and in the IGR upstream of *ssaR* (Figure 3). Functional interactions were confirmed for *sseA* and *ssaR* in subsequent reporter experiments described below.

### Identification of a functional DNA element for SsrB binding

**Figure 4:**
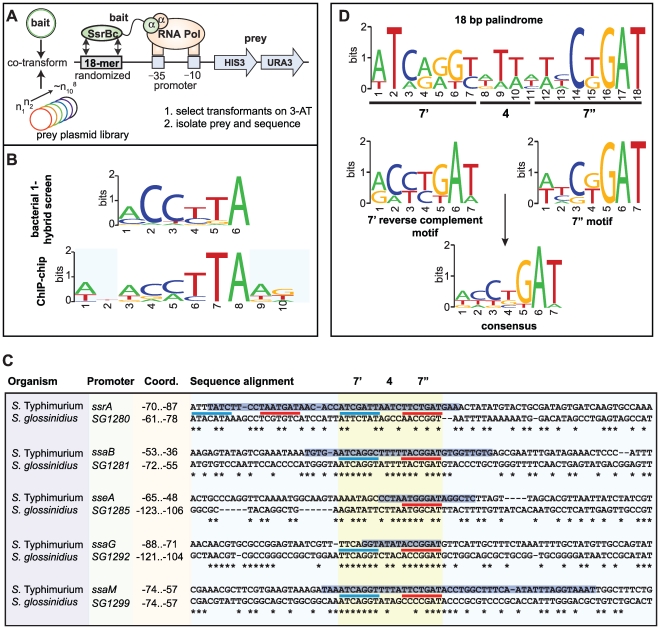
Identification of a conserved palindrome in *S.* Typhimurium SPI-2 and *S. glossinidius* SSR-3 promoters. (A) Schematic representation of the bacterial one-hybrid screen for functional SsrB binding sequences. (B) Motif logos generated independently from the bacterial one-hybrid screen and ChIP-on-chip data show similar but degenerate consensus along 6 nucleotides. (C) Aligned promoter regions from the *S.* Typhimurium SPI-2 island and *S. glossinidius* SSR-3 island containing the putative SsrB DNA recognition palindrome. Coordinates indicate position of the conserved palindrome with respect to the translational start site of the downstream gene. The 7′-4-7″ tail-to-tail palindrome sequences are underlined in blue (7′ site) and red (7″ site), aligned and highlighted in yellow. Asterisks indicate conserved nucleotides between *S*. Typhimurium and *S. glossinidius*. The positions of SsrB footprints protected from DNase1 [25],[26] are shown as black text highlighted with a blue horizontal bar. (D) Consensus motif logos for the palindrome sequences from the SPI-2 promoters shown in (C).

Previous attempts by others to identify a conserved SsrB DNA recognition motif have been unsuccessful. To overcome this, we employed a bacterial one-hybrid screen originally developed to define binding site preferences for eukaryotic transcription factors [27]. We fused the DNA binding domain of SsrB (SsrBc) to the α-subunit of RNA polymerase and screened a prey library of ∼10^8^ DNA molecules previously counter-selected against self-activation (Figure 4A). We used the PhoP response regulator from *E. coli* as a control because a DNA recognition sequence for it was known [28]. Bait-prey combinations surviving selection on medium lacking histidine were purified, and preys were sequenced and analyzed using the motif-finding program MEME [29]. From 189 unique sequences isolated for SsrBc, over 80% contained a degenerate consensus motif, mCCyTA (Figure 4B). In control screens with the PhoP-αNTD fusion, the PhoP box sequence (G/T)GTTTA was identified in 11% of sequenced preys (12/109, data not shown) but this sequence was never captured by SsrB-αNTD and vice versa, demonstrating specificity of the bacterial one-hybrid system for prokaryotic regulatory proteins.

Next, we examined our ChIP-on-chip data for the presence of a conserved regulatory motif. We extracted sequence data from the local maxima of the 256 binding peaks and analyzed the sequences with the computational program MDscan [30]. Using the highest-ranking probes to generate an initial prediction followed by lower-ranking probes for refinement, this analysis identified motifs that represented either the forward or the reverse complement of the consensus sequence ACmTTA, which shares consensus with the motif identified in the bacterial one-hybrid screen (Figure 4B). We identified variations of this motif within footprinted regions of SsrB-regulated promoters [25],, however sequence degeneracy made it difficult to precisely map the input functions.

### Identification of a functional DNA palindrome conserved in *S.* Typhimurium SPI-2 and the *Sodalis glossinidius* SSR-3 region

The analysis of regulatory evolution is particularly challenging because it is difficult to distinguish neutral stochastic change from functional divergence. To solve this problem in the context of mapping the SsrB binding element, we used comparative genomics to search for conserved promoter architecture in another organism with a similar genomic island to *Salmonella* SPI-2. The tsetse fly endosymbiont *Sodalis glossinidius* contains the *Sodalis* Symbiosis Region-3 (SSR-3) that is similar in content and synteny with the *S. enterica* SPI-2 locus [31]. Gene conservation includes the entire T3SS structural module extending to the regulatory genes *ssrA*-*ssrB* and all other genes except the effectors *sseF* and *sseG*, which are not present in *Sodalis* SSR-3. We aligned the sequences of the five mapped promoters in SPI-2 with the orthologous SSR-3 regions to identify local conservation. Highly conserved sites within the promoters were restricted to regions previously footprinted by SsrB [25],[26], whereas adjacent sequence showed substantial drift (Figure 4C). Within the conserved sites we identified a heptameric sequence in 7-4-7 tail-to-tail architecture that created an 18-bp degenerate palindrome. This palindrome was found in all SPI-2 and SSR-3 T3SS promoters with the exception of the *sseA* promoter that had only one reasonably well-conserved heptamer in the footprinted region (Figure 4C and Figure S1). Interestingly, two copies of the palindrome occur upstream of the *ssrA-ssrB* operon in *S.* Typhimurium within the same footprint, and the conservation of either site in *Sodalis* was weak. Evaluation of the heptamer motif in the palindrome showed high similarity to the motifs identified by the bacterial one-hybrid screen and the ChIP-on-chip experiments (Figure 4B and 4D), giving us confidence that we had identified the major recognition module for transcriptional input by SsrB. In accord with a previous observation [26], there was not a strict requirement in the spacing between the SsrB binding site and the downstream transcriptional start site.

### SsrB directs transcriptional input using a flexible palindrome architecture

**Figure 5:**
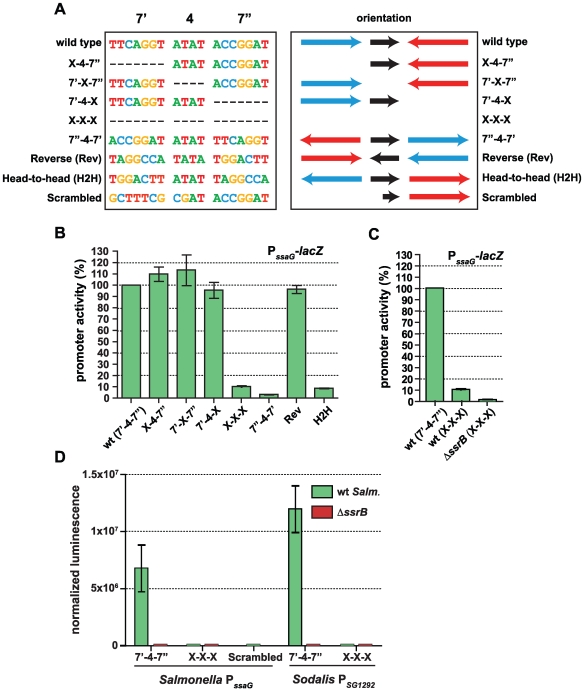
The SsrB binding motif architecture is flexible and conserved in *Sodalis*. (A) The sequences of the wild-type SsrB binding motif and mutated permutations from the *ssaG* promoter are shown. The orientation of the 7-bp half sites are indicated, where the blue arrow represents the 7′ site and the red arrow represents the 7″ site as identified in Figure 4. The orientation of the 4-bp spacer sequence is represented as a black arrow. The colours of the bases are used to distinguish the different deoxyribonucleotides. (B) Transcriptional reporter data for the wild-type *cis*-regulatory input (wt) and the seven permutations as defined in (A). Promoter activity is shown at 5 h as measured by β-galactosidase assays normalized to promoter activity from the wild-type reporter. (C) Comparison of *ssaG* promoter activity between wild-type *S*. Typhimurium and an *ssrB* mutant. β-galactosidase assays were conducted for the P*ssaG* X-X-X reporter in both wt and Δ*ssrB* cells. Data represents the promoter activity as a percent of wild type. (D) The conserved palindrome sequence from *Sodalis glossinidius* is functional. Transcriptional reporters that either contained (7′-4-7″) or lacked (X-X-X) the 18-bp palindrome sequence were constructed from evolved orthologous promoters in *Salmonella* (P*ssaG*) and *Sodalis* (P*SG1292*). An additional reporter that mutated the bases in the left heptamer to those never occurring in the consensus matrix (scrambled) was also constructed. Reporters were transformed into wild-type *Salmonella* and an *ssrB* mutant and tested for transcriptional activity. Luminescence data was normalized to the OD of the culture. Shown are the means with standard deviation for three separate experiments.

The presence of a conserved palindrome sequence in SPI-2 promoters and in related sequences from the endosymbiont *S. glossinidius* suggested that regulatory input by SsrB was through a palindrome sequence architecture. However, other lines of evidence suggested that the recognition site architecture was flexible in nature: (*i*) our bacterial one-hybrid screen isolated functional single hexamer sequences, (*ii*) the SsrB footprint at the naturally evolved *sseA* promoter within SPI-2 [26] contained only one reasonably well-conserved heptamer, and (*iii*) degenerate or non-ideal palindromes exist in the genome. In order to deconstruct this architecture, we designed a set of experiments to test the palindrome's tolerance to mutation. We chose the *ssaG* promoter for these experiments because 16 of 18 bases were identical between SPI-2 and SSR-3 from *S. glossinidius*, differing only in the 4-bp spacer between heptamers (Figure 4C). We mutated the palindrome in a series of transcriptional reporters that were otherwise identical to the evolved *ssaG* promoter (Figure 5A) and promoter activity was compared to that of a wild-type palindrome sequence. Variants in which the first half-site (7′) or second half-site (7″) was deleted produced similar transcriptional activity to the wild-type palindrome, verifying that a single well-conserved heptamer is sufficient for transcriptional input under these experimental conditions (Figure 5B). Deletion of the 4-bp spacer sequence between the heptamers - the most degenerate element of the palindrome - also generated wild-type promoter activity. However, the orientation of individual heptamers was essential for transcriptional input since rewiring the palindrome in any head-to-head orientation produced negligible promoter activity. However, if the two half sites were swapped front-to-back so that they maintained tail-to-tail orientation (construct labelled “Reverse” in Figure 5), wild-type promoter activity was restored. Precise deletion of the entire 18-bp palindrome lead to ∼10% residual activity in wild-type cells, which was reduced to less than 1% in an *ssrB* mutant (Figure 5C). To determine whether the remaining 10% transcriptional activity was a result of an SsrB-dependent feed-forward mechanism or transcriptional read-through of our chromosomally integrated reporter, we constructed an ectopic deletion reporter. Assessment of reporter activity for this construct in addition to wild-type constructs in wild-type and *ssrB* mutant backgrounds showed a similar level of activity to the *ssrB* mutant (Figure 5D).

The results for the half-site deletion constructs, which retained activity similar to wild type, were unexpected. Therefore, we compared the sequences generated upon mutation against a consensus palindrome matrix generated from all SPI-2 and other identified putative elements. The 7′-4-X, X-4-7″ and 7′-X-7″ mutations introduced a number of base transitions and transversions never occurring in the matrix, however the modified 7 base pair heptamer retained 4–5 naturally-occurring bases along with the unchanged wild-type sequence in the other heptamer (Figure S2). The possibility existed that this modified heptamer, although now weaker in consensus, could still be sufficient for recruitment of a functional form of SsrB when paired with the other wild-type heptamer. To test this, we created an additional ectopic transcriptional fusion construct in which the left half (7′) of the palindrome was mutated to bases never occurring in the consensus matrix. When tested in promoter activity experiments, this reporter was unable to activate transcription and was identical to the X-X-X mutant construct (Figure 5D).

*Salmonella* SsrB and the *Sodalis* ortholog (*SG1279*) are 69% identical and 81% similar at the amino acid level. All of the critical residues in the dimerization helix and HTH motif required for specific transcriptional activity by SsrB [32] are conserved in the *Sodalis* ortholog (Figure S3). To demonstrate a functional role for the palindrome identified in *Sodalis*, we engineered luciferase transcriptional reporters that either contained (7′-4-7″) or lacked (X-X-X) the identified palindrome from the *Sodalis SG1292* promoter (*ssaG* ortholog) and transformed them into wild-type *S.* Typhimurium and an *ssrB* mutant. The transcriptional activity from a wild-type *Sodalis* palindrome sequence was high, but was completely abolished in an *ssrB* mutant and in experiments where only the palindrome sequence was precisely deleted (Figure 5D). These experiments demonstrated a functional role for the conserved palindrome in *Sodalis* and the requirement for SsrB for transcriptional activation.

### Regulatory evolution of the SPI-2 T3SS effector repertoire

**Figure 6:**
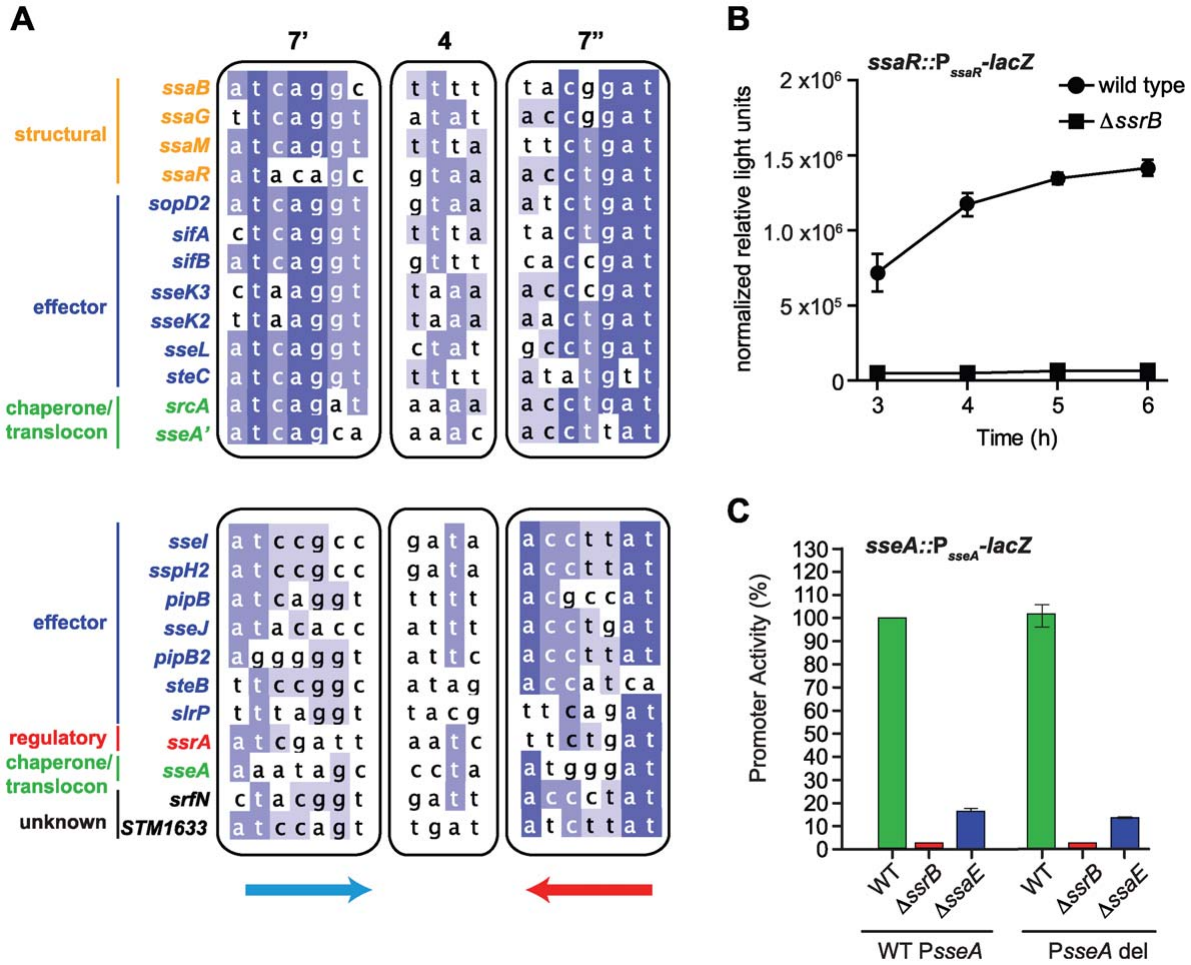
Genome-wide identification of SsrB palindrome sequences. (A) A position weight matrix was generated from all naturally-evolved palindromes in SPI-2 and used to search the genome for similar sequences. Palindromes identified in regulatory DNA that co-occurred with ChIP peaks upstream of SsrB-regulated genes were selected, aligned and binned according to those scoring >0.8 against the position weight matrix (top box) and those scoring between 0.7–0.8 (lower box). The left (7′) and right (7″) heptamers and the 4-bp spacer are displayed as a heat-map to show bases of high conservation (dark blue) from degenerate regions (light blue/white). The genes controlled by these promoters are indicated to the left of the sequences and coloured according to function: structural components of the T3SS (orange), effectors (blue), regulatory elements (red), T3SS chaperones and translocon (green), and SsrB-regulated genes of unknown function (black). (B) The high-scoring palindrome in the *ssaR* IGR is functional. A single-copy *ssaR* reporter was integrated on the chromosome and tested for functional activity in wild-type cells and in Δ*ssrB*. Promoter activity is shown as the mean with standard error from three separate experiments. (C) Functional validation of the intragenic high-scoring palindrome in the *ssaE* coding sequence. A single-copy transcriptional reporter that either contained (WT P*sseA*) or lacked (P*sseA* del) the single heptamer site in the *sseA* IGR was integrated on the chromosome in wild-type cells, or mutants lacking either *ssrB* or the *ssaE* coding sequence that removed the high-scoring intragenic palindrome (P*sseA*'). Transcriptional activity at 6 h is shown as the mean of triplicate determinations with standard error.

The above results identified the conserved, yet flexible, palindrome sequence defining DNA recognition by SsrB. To examine the extent to which regulatory evolution has been selective for this genetic architecture, we created a position weight matrix (PWM) for the five strongest palindrome sites in SPI-2 and the orthologous sites in *Sodalis* SSR-3. We then searched for representative candidates of this motif in the *S.* Typhimurium genome using the simple scoring algorithm MotifLocator [45],[46]. This analysis recovered the motifs upstream of *ssaB*, *ssaG*, *ssaM*, and *ssaR* that were used to generate the PWM. The palindrome in the *ssrA* promoter was not used to create the PWM due to its weaker consensus in the left heptamer, however, it was recovered in the computational analysis in a second group of lower-scoring motifs (Figure 6A). We identified 24 palindromes co-occurring with ChIP-on-chip peaks upstream of 24 different SsrB-regulated genes or operons. Applying a stringent threshold to the output allowed us to identify two groups - genes with high-scoring upstream palindromes (*ssaB, ssaG, ssaM, ssaR, sopD2*, *sifA*, *sifB*, *sseK2, sseK3, sseL, sseA*′, *steC,* and *srcA*) and those with medium-scoring palindromes (0.7–0.8 threshold; *ssrA, STM1633, sseI, slrP sspH2, pipB, sseJ, pipB2, srfN, sseA* and *steB*) (Figure 6A and Dataset S4) (*sseA*′ denotes the SsrB palindrome sequence upstream of *sseA* that falls within the *ssaE* CDS, while *sseA* refers to the SsrB-footprinted IGR site with only one conserved heptamer defined in Figure 4C). Remarkably, this accounted for 17 of 22 SL1344 genome-encoded effectors translocated by the SPI-2-encoded T3SS (exceptions are chromosomal *steA*, *gogB*, and *sseK*, and plasmid-encoded *spvB* and *spvC*). These genes either lacked an upstream ChIP peak above our 3-standard deviation cut-off (*sseK*) or had such a peak but did not reach statistical significance in our transcriptional profiling experiments (*steA, gogB*).

Our ChIP-on-chip data revealed three additional strong SsrB binding peaks within SPI-2: one in the IGR directly upstream of *ssaR*, a second within the CDS for *ssaJ*, and a third within the CDS for *ssaE* that would be predicted to influence transcription of the downstream effector/chaperone operon beginning with *sseA*. The analysis described above recovered SsrB palindrome sequences at the *sseA*' and *ssaR* locations prompting further validation of these sites. No palindrome was identified for the *ssaJ* interaction peak and so further characterization was not pursued. For the IGR palindrome upstream of *ssaR*, we tested both a chromosome-integrated transcriptional fusion and an autonomous episomal reporter. In wild-type cells these reporters were as active as other SPI-2 promoters, whereas promoter activity was abrogated in Δ*ssrB* cells, implicating this IGR as a functional promoter for *ssaR* (Figure 6B and Figure S4). We next tested the function of the intragenic palindrome within *ssaE* (*sseA*'). For this, we constructed a single-copy transcriptional reporter that either contained (WT P*sseA*) or lacked (P*sseA* del) the single heptamer site in the *sseA* IGR and integrated this reporter into wild-type cells and mutants lacking either *ssrB* or the *ssaE* coding sequence that removed the high-scoring intragenic palindrome *sseA*'. These experiments showed that the *sseA*' sequence contributes approximately 75% of transcriptional output at the *sseA* promoter (Figure 6C), since deleting the single heptamer in the *sseA* IGR had little effect on transcriptional output in any of the strain backgrounds. These reporter data are in line with the respective binding scores for the ChIP-on-chip interaction peaks (Figure 3) and the sequence similarity for these elements with respect to the consensus palindrome (Figure 6A and Figure 4D). Together, these data provide compelling evidence for the identity of the DNA recognition element that has been selected through evolution to co-regulate an SsrB-dependent gene program involved in adaptation to a host.

## Discussion

Horizontal gene transfer is a well-recognized mechanism of bacterial evolution that gives rise to new phenotypes due to the coordinated expression of novel genetic components [1]. A good example of this is acquisition of type III secretion by mutualists and pathogenic bacteria enabling new colonization strategies within a host [33],[34]. Evolved changes to regulatory circuitry can also give rise to phenotypic diversity at the species level [19]. In both cases, regulatory evolution is required to correctly deploy gene products during infection, yet the extent to which regulatory evolution contributes to pathogenic adaptation is only beginning to be realized [8]. The SsrA-SsrB two-component regulatory system in *S. enterica* has been the focus of our efforts to understand the significance of regulatory evolution for pathogenic adaptation. This regulatory system was co-acquired with a T3SS encoded in the SPI-2 pathogenicity island and likely contributed to immediate and gradual phenotypic diversity as new regulatory nodes were explored and acted upon by natural selection.

Extensive work has been reported on the characterization of SsrB dependent genes, including functional evaluation of genes encoded within SPI-2 in addition to genome-wide transcriptional studies [14],[15]. In this study we identified genes co-expressed under SsrB-inducing conditions and found those with strong levels of expression localized predominantly to mobile genetic elements, recently acquired genomic islands or other annotated islands. We also identified many weakly co-expressed genes, some of which may represent ancestral *Salmonella* genes recruited into the SsrB regulon like the previously reported *srfN* [18]. Some of these genes may not be directly regulated by SsrB and will require further experimental investigation.

Direct profiling of SsrB-DNA interactions using ChIP-on-chip was used to identify SsrB binding sites in the genome. This analysis identified many interactions which have not been previously described and interaction sites within coding regions of genes which may represent non-canonical functions for SsrB. Other groups have reported the existence of similar numbers of ChIP-on-chip interactions within intragenic regions for other transcription factors [35],[36] suggesting that this phenomenon is not restricted to SsrB. In light of the disparate number of microarray genes in comparison to ChIP-on-chip peaks we attempted to generate a more comprehensive picture of the SsrB regulon by combining these data sets at the operon level. In doing so we believe that the nineteen operons containing differentially expressed genes determined by microarray and containing a ChIP-on-chip peak three standard deviations above the mean captured by this analysis represent the genes directly activated by SsrB (Table 2). Those operons having a ChIP-on-chip peak directly upstream in the IGR region encompass the majority of known SsrB regulated genes while those possessing a ChIP peak within the CDS of the first gene may represent non-functional interactions that deserve follow-up experimental investigation.

The ChIP-on-chip data not only provided information on the identities of SsrB-regulated genes but also gave insight as to the identity of the SsrB recognition element specified by the interaction site sequences. The regulatory architecture governing SsrB input has been elusive despite several SsrB footprints being defined biochemically [19],[25],[26]. Our ChIP-on-chip data further suggested that SsrB binding within SPI-2 was specific, with binding peaks overlapping precisely with regions of the DNA footprinted by SsrB [25],[26]. By using a genetic screening strategy together with functional and comparative genomics, we were able to define the essential SsrB regulatory element as being an 18-bp palindrome with a conserved 7-4-7 internal organization.

In support of the palindrome as the functional entity we showed the loss of SsrB dependence as a result of deletion of this element for the *ssaG* promoter. Evaluation of the 7-4-7 palindrome in the *ssaG* promoter revealed the minimal architecture and sequence orientation required for transcriptional input. Deletion of the entire palindrome resulted in less than 1% activity in wild-type cells, an equivalent level of activity to those lacking *ssrB* entirely. A search of the *S. enterica* genome for this palindromic motif revealed candidates upstream of the previously noted SsrB dependent genes, including two additional SPI-2 sites; one IGR site upstream of *ssaR* that until now had been cryptic, and one intragenic palindrome upstream of *sseA* in the *ssaE* CDS. In both cases these input sites were found to be functional.

Although palindrome architecture was conserved upstream of SsrB-regulated genes, degenerate palindromes in which one half-site was more conserved were also functional. As a result of our mutational analyses we conclude that so long as the orientation of a single heptamer of the palindrome is conserved with respect to the downstream gene, SsrB is tolerant of degeneracy in the adjacent spacer and heptamer sequences. While we were able to identify a number of limited palindrome-like sequences from our bacterial one-hybrid screen, this tolerance in addition to the library size required to pull out an 18-bp palindrome in large numbers may explain why we isolated functional single heptamer sequences and why degenerate palindromes naturally exist in the genome. A recent report by Carroll et al, postulated that SsrB first interacts with DNA as a monomer, followed by dimerization [32]. Our findings also suggest that dimerization is likely required for transcriptional activation however strong recognition by one monomer may stabilize interaction of a second monomer with a less than ideal sequence. The finding that a flexible palindromic sequence can be selective for SsrB input raises many interesting questions around the nature of regulatory evolution. The ability to use a short functional half-site adjacent to an uncharacterized threshold level of tolerated bases would reduce the period of neutral evolution required to generate an inverted repeat sequence twice the length [37], and would limit the loss of intermediate variations to drift while a more desirable palindrome is created by regulatory evolution. For bacteria that make use of horizontal gene transfer, this could increase the tempo with which new DNA is integrated into the regulatory circuitry of the cell.

We showed that the SsrB regulatory palindrome is also present in the orthologous SSR-3 island of the endosymbiont *Sodalis glossinidius* and that the palindrome evolved in *Sodalis* can act as a *cis* regulatory input function in *Salmonella.* Thus, in addition to supporting a pathogenic lifestyle within a host in *Salmonella*, it seems probable that this common promoter architecture may direct the activation of the SSR-3 T3SS of *S. glossinidius* in its endosymbiotic relationship with the tsetse fly host, although we acknowledge this requires experimental validation. The SSR-3 region in *S. glossinidius* is fully conserved in gene synteny and content with that of SPI-2 [31], with the exception of two effector genes missing in SSR-3 (*sseF* and *sseG)* that are required to localize vacuolar *Salmonella* to the perinuclear Golgi in host cells [38],[39]. The SsrB ortholog in *S. glossinidius* is ∼30% divergent with SsrB at the protein level, initially leading us to think that they might have different binding site preferences. To the contrary, high local conservation in the promoters evolved in *Salmonella* and *Sodalis* was the crux in defining the functional SsrB input among stochastic noise. This analysis revealed strong palindrome sequence conservation in five promoters identified in SPI-2 and in the orthologous sequences in *Sodalis* SSR-3.

Among palindrome-containing promoters, the *ssrA* promoter is exceptional for two reasons: a lack of conservation between *Salmonella* and *Sodalis*, and the evolution of tandem palindromes in *Salmonella*. One possible interpretation of this divergent regulatory architecture in front of *ssrA* might relate to bacterial lifestyle. *Salmonella* may have retained or evolved SsrB input here to create a positive feedback loop on the regulatory system to rapidly adapt to the host environment during infection, similar to transcriptional surge described for the PhoP response regulator [40]. The endosymbiotic relationship of *Sodalis* with the tsetse fly - where long-term vertical transmission has ostensibly been formative in shaping regulatory circuitry at certain promoters - may obviate the need for rapid transcriptional surge, leading to regulatory drift or selection against positive feedback. With the structure of SsrB available [32] and its recognized sequence now identified, future studies will be able to build a picture of how SsrB interacts with both its target DNA, RNA polymerase and potentially other transcription factors including nucleoid associated proteins in order to direct transcription of its regulon.

In summary, this work highlights the evolutionary significance of *cis*-regulatory mutation for the adaptation of *Salmonella* to a host animal. The DNA module that choreographs SsrB-mediated pathogenic behaviour in *Salmonella* appears to have been conserved for mutualism as well, thereby shedding new light on the significance of *cis*-regulatory mutations for bacteria evolving in different ecological settings.

## Methods

### Ethics statement

All experiments with animals were conducted according to guidelines set by the Canadian Council on Animal Care. The Animal Review Ethics Board at McMaster University approved all protocols developed for this work.

### Bacterial strains and growth conditions

The *Salmonella* strain used for microarray and ChIP-on-chip analysis was *Salmonella enterica* serovar Typhimurium strain SL1344. Bacterial strains and plasmids used in this work are described in Table S1. Primer sequences used to generate constructs are available upon request. Bacteria were grown in LB medium unless otherwise indicated. Low-phosphate, low magnesium (LPM) medium was used as bacterial growth medium for microarray, ChIP-on-chip, and transcriptional reporter experiments [20]. Liquid cultures were routinely grown at 37°C with shaking. Antibiotics were added to media as follows when necessary: ampicillin (Amp, 100 µg/mL), chloramphenicol (CM, 34 µg/mL) kanamycin (Kan, 30 or 50 µg/mL), and streptomycin (SM, 50 µg/mL). NM medium was used in the bacterial one-hybrid experiments as described previously [27].

### Transcriptional profiling

Microarray experiments were conducted and analyzed as described previously [16]. cDNA was synthesized from RNA harvested from wild-type cells and an Δ*ssrB* mutant. cDNA from 2 replicate experiments was hybridized to InGen arrays and analyzed using ArrayPipe version 1.309 [21]. Probe signals underwent a foreground-background correction followed by a printTipLoess normalization by sub-grid. Duplicate spots were merged followed by averaging of the two replicates. Local intensity z scores were calculated for determination of significance.

### Genome-wide operon and island analysis

For operon analysis, *S.* Typhimurium SL1344 operons were defined as groups of genes encoded on the same strand with a maximum intergenic distance of 30-bp. Operons selected for further investigation were those possessing at least one significantly down-regulated gene from the cDNA microarray analysis of an *ssrB* mutant. A cDNA microarray score was assigned based on the average fold-change value of all genes within the operon. For ChIP-on-chip analysis, a top ChIP interaction score was defined as that of the highest scoring probe within the first gene or the intergenic region upstream of the first gene of the operon. For the analysis of regions of difference (ROD) between *S. enterica* serovar Typhimurium and *Salmonella bongori*, a reciprocal-best BLAST analysis was performed to identify orthologous genes between *S.* Typhimurium and *S. bongori*. Orthologs were defined as reciprocal best BLAST pairs with E-values less than 0.005. Comparison of gene synteny between regions encoding orthologous genes was performed to identify regions of low conservation including gene deletions and insertions. The location and names of genes flanking the comprehensive list of genomic islands is provided in Dataset S2 and were compared to those predicted using IslandViewer [41].

### Bacterial one-hybrid screen and analysis

The bacterial one-hybrid (B1H) experiments were conducted as outlined previously using a single-step selection procedure [27]. Full-length *phoP* from *E. coli* and the C-terminal domain of *ssrB* (*ssrBc*) from *S*. Typhimurium were cloned into pB1H1 to create a fusion to the αNTD of RNA polymerase. Each bait vector was transformed into *E.coli* Δ*hisB* Δ*pyrF*, purified, and then cells were transformed again with purified prey library that was previously counter-selected for self-activating preys using 5-fluoro-orotic acid. Transformants were recovered for 1 h in SOC medium, washed with NM medium supplemented with 0.1% histidine (NM + his) and allowed to grow for 2 h in this medium. Cells were washed four times with water, once with NM medium lacking histidine (NM –his), then resuspended in NM –his and plated on 150×15 mm dishes containing NM –his media supplemented with either 1 mM (for PhoP screen) or 5 mM (SsrBc screen) 3-aminotriazole. Selection was for ∼48 hours at 37°C. Individual clones were selected from plates containing <600 colonies, the prey plasmids were isolated and sent for sequencing (Macrogen USA). Sequences were parsed to extract the 18-bp prey sequence, then inputted into MEME (version 4.1.1) for motif generation [29]. MEME was run with default parameters and included searching for motifs of length 5–17 bp in either forward or reverse direction and with no limit on the number of occurrences within an input string. Motif logos were generated using Weblogo, version 2.8.2 [42].

### Chromatin immunoprecipitation-on-chip (ChIP-on-chip)

Chromatin immunoprecipitation-on-chip (ChIP-on-chip) was conducted as described previously using an SL1344 strain containing an *ssrB-3xFLAG* allele on the chromosome [19]. The primer sequences used to generate the DNA for recombination were: 5′GAG TTA CTT AAC TGT GCC CGA AGA ATG AGG TTA ATA GAG TAT GAC TAC AAA GAC CAT GAC GG3′ and 5′ATC AAA ATA TGA CCA ATG CTT AAT ACC ATC GGA CGC CCC TGG CAT ATG AAT ATC CTC CTT AG3′. This strain was generated by an allelic replacement method described previously [43] and causes lethal infection of C57BL/6 mice similar to wild-type SL1344 (Figure S5). Immunoprecipitated DNA from three experiments under SsrB-inducing conditions (LPM growth medium) and one experiment under non-inducing conditions (exponential growth in LB medium) was hybridized to a single chip printed with four whole genome arrays designed on *S. enterica* serovar Typhimurium strain SL1344 (Oxford Gene Technology, Oxford UK). Signals for each probe within an experiment were normalized to the median channel signal for the respective array. Signal ratios were obtained for both inducing and non-inducing conditions by calculating the ratio of the control probe value and experimental probe value. A final interaction score was obtained by taking the log_2_ value of the ratio between the non-inducing and inducing conditions for each probe to remove SsrB interactions that occur under non-inducing conditions. Parsing and data analyses were performed using the Python scripting language. Genome-wide ChIP-on-chip data was plotted using Circos v.0.51 [44].

### Genome-wide motif analysis

ChIP probes were ordered according to their position on the *S.* Typhimurium SL1344 genome and local maxima for ChIP interaction scores were defined as interaction peaks. Peaks with scores greater than three standard deviations from the mean probe signal were considered significant ChIP interaction peaks and were ranked in order of descending interaction score. The sequence of the top-scoring probe for each peak was exported to a text file and used for analysis by MDscan [30]. The background parameter was run with output generated by the included genomebg program from the *S.* Typhimurium SL1344 genome sequence. The initial motif was generated from sequences from the top ten SsrB interaction peaks and refined using the top 25 peak sequences.

To identify instances of the palindrome motif in *S.* Typhimurium, ten 18-bp 7-4-7 palindromic motifs in the promoters of the SPI-2 genes *ssrA*, *ssaB*, *ssaG*, *ssaM*, *ssaR* and their orthologous SSR-3 genes were used as input for MDScan to identify a consensus motif and to determine the position specific scoring matrix (PWM). This PWM was used with MotifLocator to identify instances of this motif in the S. Typhimurium SL1344 genome. A background file specific to SL1344 was generated using the associated script called CreateBackgroundModel. A stringent threshold value of 0.8 was used [45],[46].

### Transcriptional reporter experiments

Transcriptional fusions to *lacZ* for the *ssaG* and *sseA* promoter palindrome analysis were generated using chemically synthesized double-stranded DNA (Genscript Corp). Promoter DNA was ligated into pIVET5n, then the plasmid was subsequently conjugated into SL1344 to generate single-copy transcriptional fusions integrated on the chromosome as described previously [20]. Luciferase reporter constructs for the *Sodalis glossinidius SG1292* and *ssaR* promoters were generated by PCR amplification of promoter regions from genomic DNA templates. The luciferase reporter construct for the P*ssaG* scrambled substitution was created by PCR product splicing via overlap extension using the existing *ssaG* promoter cloning primers and two additional internal primers containing the desired mutation sequence (DTM0061R, 5′CGC GAA AGC AAC GAT TAC TCC GGC GCA CG3′ and DTM0061.1F, 5′GAG TAA TCG TTG CTT TCG CGA TAC CGG ATG TTC ATT GCT TTC TA3′). This DNA was ligated into pCS26 and transformed into SL1344 to generate plasmid-based reporters. Overnight cultures of *Salmonella* were sub-cultured into LPM medium and grown with shaking for 7 h. Samples were removed hourly to measure β-galactosidase activity via a chemiluminescence-based assay as described previously [20] or luminescence directly from cultures (EnVision, Perkin-Elmer). Output was relative light units (RLU) normalized to OD600. Each experiment was performed in triplicate then averaged. Reporter activity from mutant and rewired promoters was normalized to that from wild-type promoters.

### Data deposition

All ChIP-on-chip data can be retrieved from the NCBI Gene Expression Omnibus at http://www.ncbi.nlm.nih.gov/geo/query/acc.cgi?acc=GSE20192. Data files for viewing in Artemis (http://www.sanger.ac.uk/Software/Artemis/) are available upon request.

## Supporting Information

Figure S1 *S.* Typhimurium SL1344 SPI-2 and *S. glossinidius str. ‘morsitans’* SSR-3 motifs. Sequence logos of the half-site (7′ and 7″) and full-length (7′-4-7″) palindrome motif identified within the promoter regions of SPI-2 in *S.* Typhimurium and SSR-3 T3SS in *S. glossinidius*. ‘Combined’ refers to the consensus sequence based on the single left and right heptamer sequences within a given organism. ‘Merged’ refers to the consensus sequence for individual left and right heptamers from both *Salmonella* and *Sodalis*.
(0.14 MB PDF)

Figure S2 Frequency matrix analysis of SsrB palindromes. Presented is a frequency matrix of nucleotides occurring at each position of the 18-bp position in the 24 identified palindromes in *S.* Typhimurium SL1344. The 18-bp palindrome is shown as two 9-bp sequences with positions (-2) and (-1) referring to the spacer and positions 1–7 referring to the heptamer as indicated at the top of the figure. Matrices are shown for the left and right components of the palindrome in addition to a combined matrix. Preferred, tolerated and disliked nucleotides are assigned based on frequency thresholds of 0.1 and 0.3 and are indicated by green, blue, and red respectively. Sequences for generated constructs are shown at the bottom of the figure and sequence identities with respect to the combined matrix are noted to the right of each construct sequence. The two possible permutations of the mutated palindrome are shown for the ssaG 7-X-7 construct.
(0.01 MB PDF)

Figure S3 Alignment of *Salmonella* SsrB and the ortholog from *Sodalis glossinidius*. Full length protein sequences of SsrB from *S.* Typhimurium strain SL1344 (SL1325) and the *Sodalis* ortholog (SG1279) were aligned with ClustalW. The helices are labeled according to the structure of SsrBc as described by Carroll et al. 2009. Helices H2 and H3 make up the helix-turn-helix DNA binding motif (H168-K191) and the dimerization helix is H4. The conserved aspartic acid residue (D56) and other residues shown to be important for transcriptional activity by Carroll et al. 2009 are indicated in red.
(0.06 MB PDF)

Figure S4 Episomal transcriptional reporter for the *ssaR* palindrome. Luminescence production from a P*ssaR-lux* transcriptional fusion indicates an active, SsrB-dependent promoter at this location, in accord with SsrB binding to this location *in vivo*. Shown are data (mean with standard deviation) from triplicate determinations from three separate experiments. Luminescence was normalized to the optical density of the culture.
(0.12 MB PDF)

Figure S5 Mouse virulence data for the *ssrB-FLAG* SL1344 strain. Groups of C57BL/6 mice were infected by oral gavage with 10^6^ colony forming units of wild-type *S.* Typhimurium strain SL1344 or SL1344 containing an allelic replacement of ssrB-FLAG. Mice were monitored for endpoint and sacrificed when they had lost 20% of their initial body weight. The percent of mice surviving on each day after infection is shown. There is no statistical difference between the groups.
(0.13 MB PDF)

Table S1 List of strains and plasmids used in this study.
(0.10 MB PDF)

Dataset S1 Significantly down-regulated microarray genes (ratio of Δ*ssrB/wt*).
(0.06 MB XLS)

Dataset S2 Genomic islands and non-conserved regions (between *S. enterica sv*. Typhimurium and *Salmonella bongori*) referenced in this study.
(0.04 MB XLS)

Dataset S3 Top-scoring SsrB ChIP-on-chip interaction peaks.
(0.10 MB XLS)

Dataset S4 Data and motif analysis for SPI-2 and associated genes.
(0.04 MB XLS)
