# Supplementary figures and images for: Identification of the Regulatory Logic Controlling Salmonella Pathoadaptation by the SsrA-SsrB Two-Component System
Source: PLoS Genet. 2010 Mar 12;6(3):e1000875. doi: 10.1371/journal.pgen.1000875 (PMC2837388; doi:10.1371/journal.pgen.1000875)

**A**

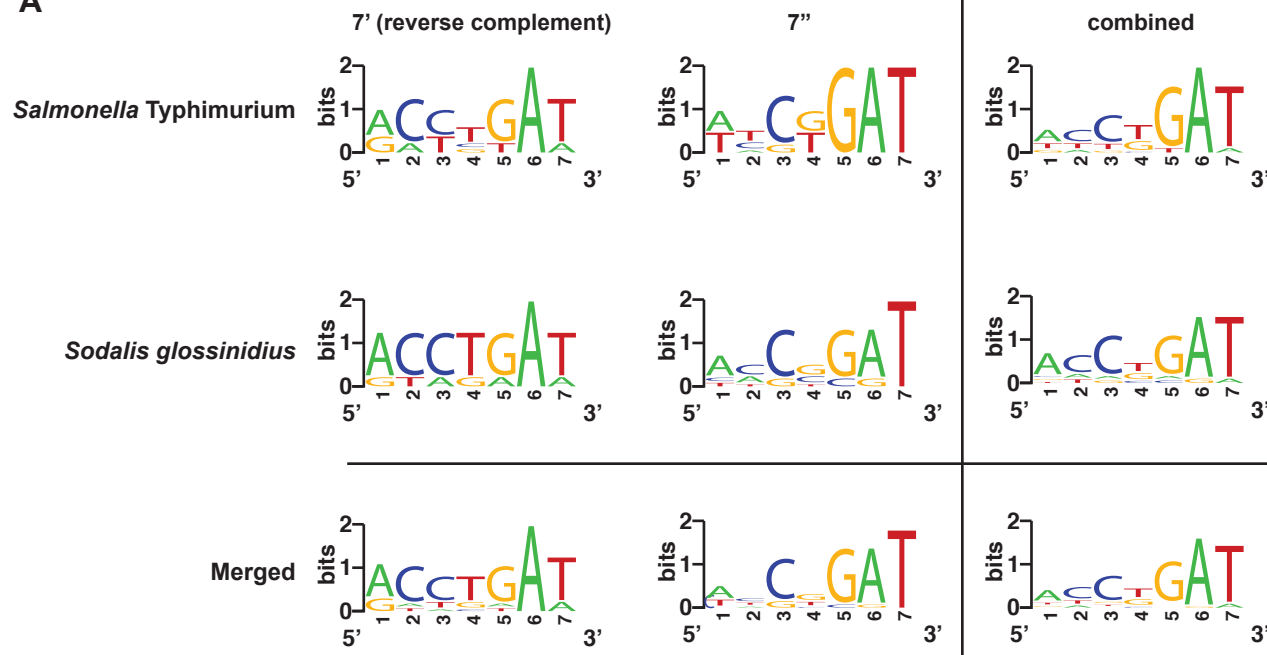

**B**

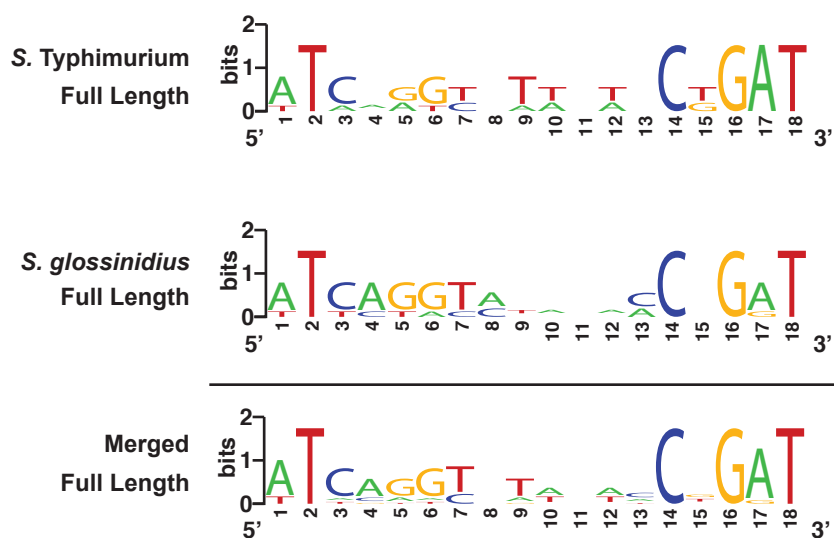

Tomljenovic et al. Figure S1

Supplement: Figure S1 — S. Typhimurium SL1344 SPI-2 and S. glossinidius str. ‘morsitans’ SSR-3 motifs. Sequence logos of the half-site (7′ and 7″) and full-length (7′-4-7″) palindrome motif identified within the promoter regions of SPI-2 in S. Typhimurium and SSR-3 T3SS in S. glossinidius. ‘Combined’ refers to the consensus sequence based on the single left and right heptamer sequences within a given organism. ‘Merged’ refers to the consensus sequence for individual left and right heptamers from both Salmonella and Sodalis. (0.14 MB PDF) [file pgen.1000875.s001.pdf]

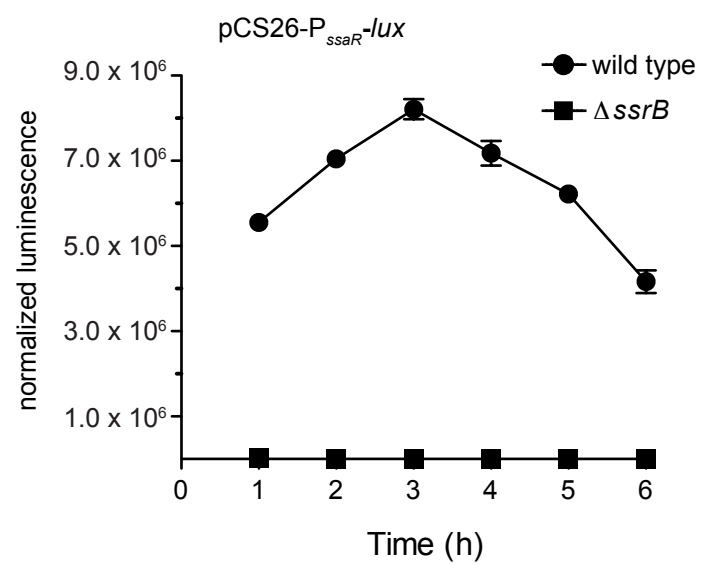

Tomljenovic et al. Figure S4

Supplement: Figure S4 — Episomal transcriptional reporter for the ssaR palindrome. Luminescence production from a PssaR-lux transcriptional fusion indicates an active, SsrB-dependent promoter at this location, in accord with SsrB binding to this location in vivo. Shown are data (mean with standard deviation) from triplicate determinations from three separate experiments. Luminescence was normalized to the optical density of the culture. (0.12 MB PDF) [file pgen.1000875.s004.pdf]

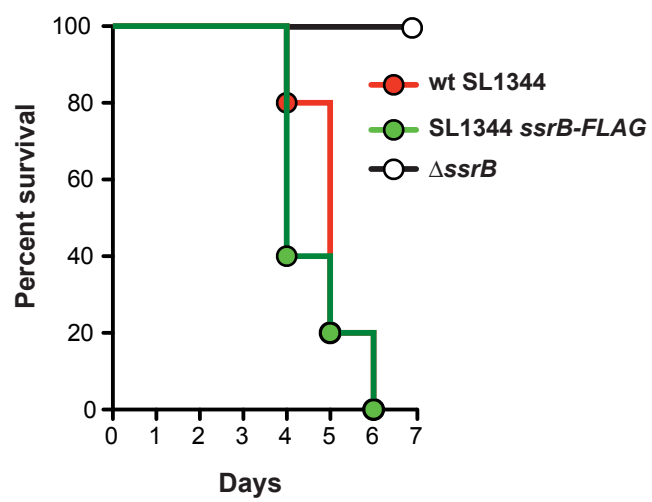

Tomljenovic et al. Figure S5

Supplement: Figure S5 — Mouse virulence data for the ssrB-FLAG SL1344 strain. Groups of C57BL/6 mice were infected by oral gavage with 106 colony forming units of wild-type S. Typhimurium strain SL1344 or SL1344 containing an allelic replacement of ssrB-FLAG. Mice were monitored for endpoint and sacrificed when they had lost 20% of their initial body weight. The percent of mice surviving on each day after infection is shown. There is no statistical difference between the groups. (0.13 MB PDF) [file pgen.1000875.s005.pdf]
