# Supplementary material for: Identification of the Regulatory Logic Controlling Salmonella Pathoadaptation by the SsrA-SsrB Two-Component System
Source: PLoS Genet. 2010 Mar 12;6(3):e1000875. doi: 10.1371/journal.pgen.1000875 (PMC2837388; doi:10.1371/journal.pgen.1000875)
Supplement: Figure S2 — Frequency matrix analysis of SsrB palindromes. Presented is a frequency matrix of nucleotides occurring at each position of the 18-bp position in the 24 identified palindromes in S. Typhimurium SL1344. The 18-bp palindrome is shown as two 9-bp sequences with positions (-2) and (-1) referring to the spacer and positions 1–7 referring to the heptamer as indicated at the top of the figure. Matrices are shown for the left and right components of the palindrome in addition to a combined matrix. Preferred, tolerated and disliked nucleotides are assigned based on frequency thresholds of 0.1 and 0.3 and are indicated by green, blue, and red respectively. Sequences for generated constructs are shown at the bottom of the figure and sequence identities with respect to the combined matrix are noted to the right of each construct sequence. The two possible permutations of the mutated palindrome are shown for the ssaG 7-X-7 construct. (0.01 MB PDF) [file pgen.1000875.s002.pdf]

Preferred (>= 0.3)
Tolerated (0.1 < x < 0.3)
Disliked (<=0.1)

LEFT HEPTAMER*

NUC/POSITION	-2 (Spacer)	-1 (Spacer)	1 (Heptamer)	2	3	4	5	6	7
A	0.583	0.38	0.67	0.04	0	0.04	0.04	0.92	0.13
T	0.333	0.29	0.04	0.08	0.21	0.54	0.25	0.04	0.75
C	0.042	0.25	0	0.71	0.79	0.08	0.04	0.04	0
G	0.042	0.08	0.29	0.17	0	0.33	0.67	0	0.13

RIGHT HEPTAMER

NUC/POSITION	-2	-1	1	2	3	4	5	6	7
A	0.458	0.42	0.71	0.21	0.04	0.04	0	0.92	0.04
T	0.542	0.38	0.21	0.25	0	0.67	0.29	0.04	0.96
C	0	0.13	0.04	0.54	0.88	0.17	0.04	0.04	0
G	0	0.08	0.04	0	0.08	0.13	0.67	0	0

COMBINED

NUC/POSITION	-2	-1	1	2	3	4	5	6	7
A	0.521	0.4	0.69	0.13	0.02	0.04	0.02	0.92	0.08
T	0.438	0.33	0.13	0.17	0.1	0.6	0.27	0.04	0.85
C	0.021	0.19	0.02	0.63	0.83	0.13	0.04	0.04	0
G	0.021	0.08	0.17	0.08	0.04	0.23	0.67	0	0.06

CONSTRUCTS

Construct		-2	-1	1	2	3	4	5	6	7	Entire Identity (9)	Heptamer Identity (7)
ssaG X-4-7	Left (7')*	A	T	A	A	C	G	A	T	T	78%	71%
	Right (7'') wt	A	T	A	C	C	G	G	A	T	100%	100%
ssaG 7-4-X	Left (7') wt *	A	T	A	C	C	T	G	A	A	89%	86%
	Right (7'')	A	T	G	T	T	C	A	T	T	67%	57%
ssaG 7-X-7	Left (7') wt *	A	T	A	C	C	T	G	A	A	89%	86%
	Right (7'')	C	G	G	A	T	G	T	T	C	44%	57%
ssaG 7-X-7	Left (7') *	C	T	G	A	A	A	A	C	G	33%	29%
	Right (7'') wt	A	T	A	C	C	G	G	A	T	100%	100%
ssaG scrambled	Left (7') *	C	G	C	G	A	A	A	G	C	0%	0%
	Right (7'') wt	A	T	A	C	C	G	G	A	T	100%	100%
sseA	Left (7') wt *	G	G	G	C	T	A	T	T	T	44%	57%
	Right (7'') wt	T	A	A	T	G	G	G	A	T	89%	86%
sseA del	Left (7') wt *	G	G	G	C	T	A	T	T	T	44%	57%
	Right (7'')	T	A	A	G	G	C	T	C	T	67%	57%

* reverse complement

Tomljenovic et al. Figure S2
