# Supplementary material for: Identification of the Regulatory Logic Controlling Salmonella Pathoadaptation by the SsrA-SsrB Two-Component System
Source: PLoS Genet. 2010 Mar 12;6(3):e1000875. doi: 10.1371/journal.pgen.1000875 (PMC2837388; doi:10.1371/journal.pgen.1000875)
Supplement: Table S1 — List of strains and plasmids used in this study. (0.10 MB PDF) [file pgen.1000875.s006.pdf]

Table S1

Strain or plasmid	Genotype or description	Reference
**Strains**		
DH5α	*supE44* D*lacu169* (f80 *lacZDM15*) *hsdR17 recA1 endA1 gyrA96 thi-1 relA1*	Lab strain
DH5α λ*pir*	*supE44* D*lacu169* (f80 *lacZDM15*) *hsdR17 recA1 endA1 gyrA96 thi-1 relA1* λ*pir*	Lab strain
E. coli SM10 λ*pir*	*thi recA thr leu tonA lacY supE* RP4–2–Tc::*Mu* λ*pir*	Lab strain
E. coli B1H	uso F' *pyrF hisB*; bacterial one-hybrid host strain	[1]
SL1344	Wild-type *S. enterica* sv. Typhimurium, SmR	[2]
SL1344 *ssrB*::*aphT*	Marked deletion of *ssrB*, KanR	[3]
SL1344 Δ*ssrB*	Unmarked, in-frame deletion of *ssrB*	This work
SL1344 Δ*ssaE*	Unmarked, in-frame deletion of *ssaE*	This work
SL1344 *ssrB::3FLAG-kan*	Encodes chromosomally FLAG-tagged *ssrB,* KanR	[4]
SL1344 P*ssaG*::pIVET-P*ssaG*	Merodiploid containing integrated P*ssaG-tnpR-lacZ* reporter	This work
SL1344 P*ssaG*::pIVET-P*ssaG*-X47	Merodiploid containing integrated P*ssaG*-X47-*tnpR-lacZ* reporter	This work
SL1344 P*ssaG:*:pIVET-P*ssaG*-7X7	Merodiploid containing integrated P*ssaG*-7X7-*tnpR-lacZ* reporter	This work
SL1344 P*ssaG:*:pIVET-P*ssaG*-74X	Merodiploid containing integrated P*ssaG*-74X-*tnpR-lacZ* reporter	This work
SL1344 P*ssaG:*:pIVET-P*ssaG*-XXX	Merodiploid containing integrated P*ssaG*-XXX-*tnpR-lacZ* reporter	This work
SL1344 P*ssaG:*:pIVET-P*ssaG*-H2H	Merodiploid containing integrated P*ssaG*-H2H-*tnpR-lacZ* reporter	This work
SL1344 P*ssaG:*:pIVET-P*ssaG*-Rev	Merodiploid containing integrated P*ssaG*-Rev-*tnpR-lacZ* reporter	This work
SL1344 P*ssaG:*:pIVET-P*ssaG*-7"-4-7'	Merodiploid containing integrated P*ssaG*-7"-4-7'-*tnpR-lacZ* reporter	This work
SL1344 P*sseA*::pIVET-P*sseA*	Merodiploid containing integrated P*sseA-tnpR-lacZ* reporter	This work
SL1344 P*sseA*::pIVET-P*sseA*del	Merodiploid containing integrated P*sseA*del-*tnpR-lacZ* reporter	This work
SL1344 Δ*ssrB* P*sseA*::pIVET-P*sseA*	*ssrB* mutant, merodiploid containing integrated P*sseA-tnpR-lacZ* reporter	This work
SL1344 Δ*ssrB* P*sseA*::pIVET-P*sseA*del	*ssrB* mutant, merodiploid containing integrated P*sseA*del-*tnpR-lacZ* reporter	This work
SL1344 Δ*ssaE* P*sseA*::pIVET-P*sseA*	*ssaE* mutant, merodiploid containing integrated P*sseA-tnpR-lacZ* reporter	This work
SL1344 Δ*ssaE* P*sseA*::pIVET-P*sseA*del	*ssaE* mutant, merodiploid containing integrated P*sseA-tnpR-lacZ*del reporter	This work
SL1344 P*ssaR*::pIVET-P*ssaR*	Merodiploid containing integrated P*ssaR-tnpR-lacZ* reporter	This work
**Plasmids**		
pB1H1	Bacterial one-hybrid bait plasmid, CmR	[1]
pB1H1-*ssrBc*	Bait plasmid carrying the C-terminal domain of *ssrB* (*ssrBc*), CmR	This work
pB1H1-*phoP*	Bait plasmid carrying *E. coli phoP*, CmR	This work
pH3U3	Bacterial one-hybrid prey plasmid, KanR	[1]
pIVET5n	*tnpR-lacZ*, *sacB*, R6K ori, *bla*, AmpR	[5]
pCS26	pSC101 *ori, luxCDABE,* KanR	[6]
pIVET-P*ssaG*	*ssaG* promoter fused to *tnpR-lacZ* in pIVET5n, AmpR	This work
pIVET-P*ssaG*-X47	*ssaG* promoter with "X-4-7" mutation fused to *tnpR-lacZ* in pIVET5n, AmpR	This work
pIVET-P*ssaG*-7X7	*ssaG* promoter with "7-X-7" mutation fused to *tnpR-lacZ* in pIVET5n, AmpR	This work
pIVET-P*ssaG*-74X	*ssaG* promoter with "7-4-X" mutation fused to *tnpR-lacZ* in pIVET5n, AmpR	This work
pIVET-P*ssaG*-XXX	*ssaG* promoter with "X-X-X" mutation fused to *tnpR-lacZ* in pIVET5n, AmpR	This work
pIVET-P*ssaG*-H2H	*ssaG* promoter with "H2H" mutation fused to *tnpR-lacZ* in pIVET5n, AmpR	This work
pIVET-P*ssaG*-Rev	*ssaG* promoter with 'Rev" mutation fused to *tnpR-lacZ* in pIVET5n, AmpR	This work
pIVET-P*ssaG*-7"-4-7'	*ssaG* promoter with "7"-4-7'" mutation fused to *tnpR-lacZ* in pIVET5n, AmpR	This work
pIVET-P*sseA*	*sseA* promoter fused to *tnpR-lacZ* in pIVET5n, AmpR	This work
pIVET-P*sseA*del	*sseA* promoter with motif deletion mutation fused to *tnpR-lacZ* in pIVET5n, AmpR	This work
pIVET-P*ssaR*	*ssaR* promoter fused to *tnpR-lacZ* in pIVET5n, AmpR	This work

pCS26-P*ssaG*	*ssaG* promoter fused to *luxCDABE* in pCS26, KanR	This work
pCS26-P*ssaG*-XXX	*ssaG* promoter with “X-X-X” deletion fused to *luxCDABE* in pCS26, KanR	This work
pCS26-P*SG1292*	*Sodalis glossinidius SG1292* promoter fused to *luxCDABE* in pCS26, KanR	This work
pCS26-P*SG1292*-XXX	*Sodalis glossinidius SG1292* promoter with “X-X-X” deletion fused to *luxCDABE* in pCS26, KanR	This work
pCS26-P*ssaR*	*ssaR* promoter fused to *luxCDABE* in pCS26, KanR	This work
pCS26-PssaG mut(1-9)	Scrambled *ssaG* promoter fused to *luxCDABE* in pCS26, KanR	This work

Reference list for Table S1

1. Meng X, Brodsky MH, Wolfe SA (2005) A bacterial one-hybrid system for determining the DNA-binding specificity of transcription factors. Nat Biotechnol 23: 988-994.

2. Wray C, Sojka WJ (1978) Experimental *Salmonella typhimurium* infection in calves. Res Vet Sci 25: 139-143.

3. Knodler LA, Celli J, Hardt WD, Vallance BA, Yip C, et al. (2002) *Salmonella* effectors within a single pathogenicity island are differentially expressed and translocated by separate type III secretion systems. Mol Microbiol 43: 1089-1103.

4. Duong N, Osborne S, Bustamante VH, Tomljenovic AM, Puente JL, et al. (2007) Thermosensing coordinates a cis-regulatory module for transcriptional activation of the intracellular virulence system in *Salmonella enterica* serovar Typhimurium. J Biol Chem 282: 34077-34084.

5. Brown NF, Vallance BA, Coombes BK, Valdez Y, Coburn BA, et al. (2005) *Salmonella* Pathogenicity Island 2 Is Expressed Prior to Penetrating the Intestine. PLoS Pathog 1: e32.

6. Beeston AL, Surette MG (2002) pfs-dependent regulation of autoinducer 2 production in *Salmonella enterica* serovar Typhimurium. J Bacteriol 184: 3450-3456.
