# Supplementary material for: Identification of the Regulatory Logic Controlling Salmonella Pathoadaptation by the SsrA-SsrB Two-Component System
Source: PLoS Genet. 2010 Mar 12;6(3):e1000875. doi: 10.1371/journal.pgen.1000875 (PMC2837388; doi:10.1371/journal.pgen.1000875)
Supplement: Figure S3 — Alignment of Salmonella SsrB and the ortholog from Sodalis glossinidius. Full length protein sequences of SsrB from S. Typhimurium strain SL1344 (SL1325) and the Sodalis ortholog (SG1279) were aligned with ClustalW. The helices are labeled according to the structure of SsrBc as described by Carroll et al. 2009. Helices H2 and H3 make up the helix-turn-helix DNA binding motif (H168-K191) and the dimerization helix is H4. The conserved aspartic acid residue (D56) and other residues shown to be important for transcriptional activity by Carroll et al. 2009 are indicated in red. (0.06 MB PDF) [file pgen.1000875.s003.pdf]

```
SG1279      MNNYKILLVDDHELIINGIINLLEPYPRFKIVAHIDDGLAVYNQCRIHEPDILVLDLGLP 60
SL1325      MKEYKILLVDDHEIIINGIMNALLPWPHFKIVEHVKNGLEVYNACCAYEPDILILDLSLP 60
            *::**********:*****:* * *:*:**** *:.:** *** *  :*****:***.**

SG1279      GINGLDLIPQLRSRWPQMSILAYTAHTEEYMAVRTLAAGALGYVLKNSRQQVLLAALQTV 120
SL1325      GINGLDIIPQLHQRWPAMNILVYTAYQQEYMTIKTLAAGANGYVLKSSSQQVLLAALQTV 120
            ******:****:.*** *.**.***: :***:::****** *****.* ***********

SG1279      AVNKSYIDPALNREMIHTALSMEAENQELLTPRERQILKLIADGYTNRLIAEQLCISVKT 180
SL1325      AVNKRYIDPTLNREAILAELNADTTNHQLLTLRERQVLKLIDEGYTNHGISEKLHISIKT 180
            **** ****:**** * : *. :: *::*** ****:**** :****: *:*:* **:**
                                             H1           H2         H3

SG1279      VETHRLNIMRKLNVHKVTELLNCSRRLGLTD- 211
SL1325      VETHRMNMMRKLQVHKVTELLNCARRMRLIEY 212
            *****:*:****:**********:**: * :
              H3              H4
```

Tomljenovic et al. Figure S3
